# Supplementary material for: Identification of Freezing-Responsive microRNAs and Their Targets in Chinese Jujube by Small RNA and Degradome Sequencing
Source: Int J Mol Sci. 2026 Sep 4;27(17):7912. doi: 10.3390/ijms27177912 (PMC13566890; doi:10.3390/ijms27177912)
Supplement: Supplementary file 1 [file ijms-27-07912-s001.zip › Supplementary Material S2/novel_miR_215.pdf]

Provisional ID	:	novel_miR_215
Score total	:	21.7
Score for star read(s)	:	3.9
Score for read counts	:	20
Score for mfe	:	0
Score for randfold	:	-2.2
Score for cons. seed	:	
Total read count	:	51
Mature read count	:	50
Loop read count	:	0
Star read count	:	1

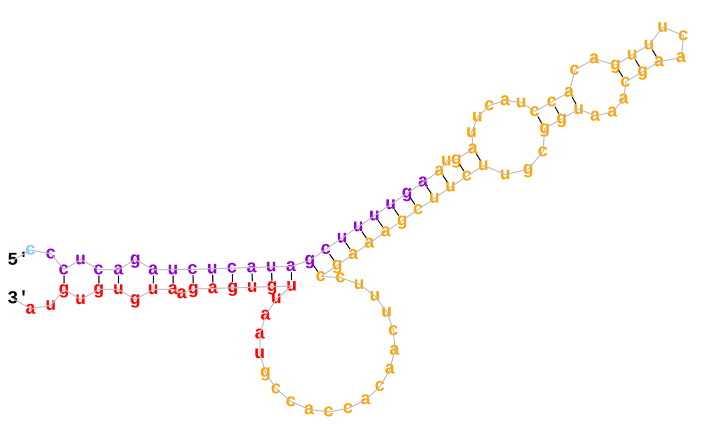

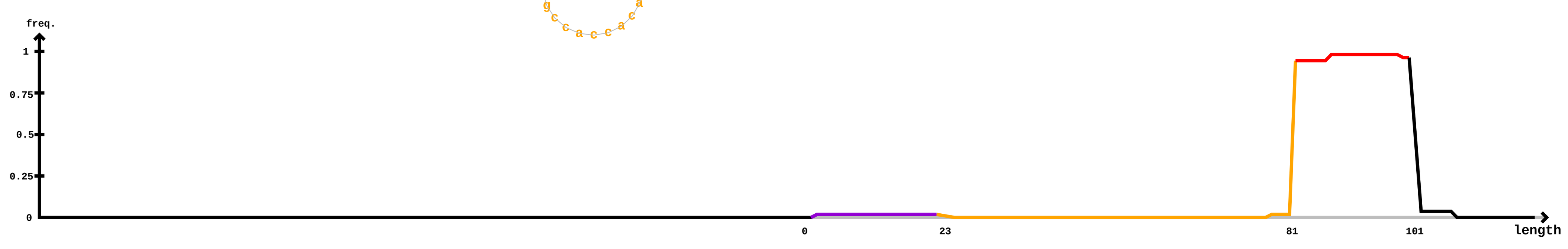


```
                                                                                                                                    Star                                                                         Mature
5'- aaaaacaaaaaucaaaauuuccauaaauaaaaagagauucuaacaagucuaagcccgguuuggagcuucauucacaaaugguuccaucuagaaaagcgaaaugcacaaaaauaaaaauauaaaaagcccucagaucucauagcuuuugaaugauucauccacaguuucaagcaaauggcguucuucgaaagccuuucaacaccaccguaauugugagaauguguguauuauguguaugguugugugugu   -3'   obs
    aaaaacaaaaaucaaaauuuccauaaauaaaaagagauucuaacaagucuaagcccgguuuggagcuucauucacaaaugguuccaucuagaaaagcgaaaugcacaaaaauaaaaauauaaaaagcccucagaucucauagcuuuugaaugauucauccacaguuucaagcaaauggcguucuucgaaagccuuucaacaccaccguaauugugagaauguguguauuauguguaugguugugugugu         exp
    ..............((((((............))))))........((((((((((.(((((((.....))).))))))))))).....))))........(((((((..(((...((((((...((.(.((..((((((((((((((((.((.....(((..(((...)))...)))...)))))))))))).................))))))).)).).)).)))))).)))..))))))))..   reads   mm   sample
    ...............................................................................................................................ccucagaucucauagcuuuuga....................................................................................................   1       0    JCK-1
    ..........................................................................................................................................................................................................accguaauugugagaaugugug.........................   1       0    LT-3
    ..............................................................................................................................................................................................................uaauugugagaaugugugua.......................   18      0    LCK-2
    ..............................................................................................................................................................................................................uaauugugagaaugugugua.......................   32      0    LCK-1
    ....................................................................................................................................................................................................................ugagaauguguguauuaugug................   2       0    LT-2
```
